# Chemotherapeutic Researches on Cancer. A Review
*"Chemotherapeutic Researches on Cancer," by A. T. Todd. M.D., M.R.C.P., Bristol; J. W. Arrowsmith Ltd., 1928. Pp. 127, Price 2s. 6d.


**Published:** 1928

**Authors:** J. A. Murray

**Affiliations:** Director of the Imperial Cancer Research Institute, London


### CHEMOTHERAPEUTIC RESEARCHES ON CANCER.*

### A REVIEW.

By J. A. Murray, M.D., B.Sc.,
*Director of the Imperial Cancer Research Institute, London.*

The recent International Conference seems to have thickened rather than cleared the fog of debate which envelops the colloidal lead treatment of cancer. It is not remarkable that this should be so, for the exact knowledge of the relation of a malignant growth to the organism bearing it is still lacking. The chorionic hypothesis of Beard and Blair Bell of the nature of cancer is far from satisfactory. The invasive and destructive appearances in the deciduate placentæ of rodents and primates is clearly much more due to the peculiar reaction of the maternal tissues in these orders than to primary properties of chorion (or trophoblast) as such. This may be inferred from the complete absence of these features in the non-deciduate placentæ (*e.g.* in the sheep), in which the villi dip into the uterine glands without entering into organic union with the maternal tissues. The conclusion is slowly gaining in cogency that the abortifacient action of lead as well as the ameliorations recorded in cancer cases are secondary to changes in the host tissues. From this point of view the lead treatment ranges itself with the various forms of radio-therapy, in which the clinical results are only intelligible as mainly consequent on an action on the supporting and nutritive stroma and not on a primary destructive action on the cancer cells themselves.

The investigations of Dr. Todd and his collaborators make a welcome objective addition to the elucidation of these intricate problems. Starting from the position taken up by Blair Bell in introducing the method, Dr. Todd has endeavoured to modify the treatment in the light of animal experiments and clinical experience in the direction of diminished toxicity of the colloid and greater uniformity in action. He brings a good deal of evidence to show that the uncontrollable toxicity of the lead-element colloids is due to their rapid passage to the ionised form, and has substituted colloidal preparations of

* "Chemotherapeutic Researches on Cancer," by A. T. Todd. M.D., M.R.C.P., Bristol; J. W. Arrowsmith Ltd., 1928. Pp. 127, Price 2s. 6d.

lead compounds, sulphide, selenide and telluride. Of these the selenide has proved the most satisfactory in his hands. In the animal experiments it is very significant that in a few animals (mice with implanted mammary carcinoma 63) recrudescence after apparently complete disappearance has occurred. This phenomenon, which is only too closely paralleled by some of the most successful human cases, is incompatible with the original hypothesis of a direct toxic action on the parenchyma cells, but quite intelligible if the action is indirect through the stroma. A remarkable and unexplained feature of the treatment of human cases is the striking analgesic action displayed. This has also been recorded for other colloidal preparations, and it would be of the greatest interest to know how it is brought about. The problem may be recommended to the physiologists as worthy of their attention.

Dr. Todd has given special prominence to the blood changes induced by these colloids. The effects in animals are employed as a preliminary test of toxicity, and in the clinical work repeated examinations of the blood are carried out to anticipate the development of the severe aplastic anæmia which is fatal to success. He has found that the administration of liver and liver extract permits of a higher dosage of colloid, and the accumulation of lead in the system is counteracted by the administration of potassium iodide.

Separate chapters are contributed on the preparation of the colloids and of the protective or stabilising substances.

What is the future outlook for the colloidal lead therapy in cancer ? If the views put forward in this review are sound, the method does not introduce a new chemotherapeutic principle. It achieves an alteration of the supporting and nutritive structures of the new growth of the same kind as that produced by X-rays and radium which may, in some cases, lead to regression or arrest of varying duration. As in so many other fields of therapeutics, *le mieux est l'ennemi du bon*, and until the possibility of a more rational direct chemotherapeutic attack is demonstrated we must surely encourage the development of any and every method which is capable of modifying the inexorable progressive march of malignant new growths.

